# Rethinking Optimal Immunogens to Face SARS‐CoV‐2 Evolution Through Vaccination

**DOI:** 10.1111/irv.70076

**Published:** 2025-01-28

**Authors:** Julià Blanco, Benjamin Trinité, Joan Puig‐Barberà

**Affiliations:** ^1^ IrsiCaixa Badalona Catalonia Spain; ^2^ Germans Trias i Pujol Research Institute (IGTP) Badalona Catalonia Spain; ^3^ CIBER de Enfermedades Infecciosas Madrid Spain; ^4^ Chair in Infectious Diseases and Immunity, Faculty of Medicine University of Vic‐Central University of Catalonia (UVic‐UCC) Vic Catalonia Spain; ^5^ Área de Investigación en Vacunas Fundación para el Fomento de la Investigación Sanitaria y Biomédica de la Comunitat Valenciana Valencia Spain

**Keywords:** immune imprinting, immunofocusing, neutralizing antibodies, RBD, spike protein, T‐cell responses

## Abstract

SARS‐CoV‐2, which originated in China in late 2019, quickly fueled the global COVID‐19 pandemic, profoundly impacting health and the economy worldwide. A series of vaccines, mostly based on the full SARS‐CoV‐2 Spike protein, were rapidly developed, showing excellent humoral and cellular responses and high efficacy against both symptomatic infection and severe disease. However, viral evolution and the waning humoral neutralizing responses strongly challenged vaccine long term effectiveness, mainly against symptomatic infection, making necessary a strategy of repeated and updated booster shots. In this repeated vaccination context, antibody repertoire diversification was evidenced, although immune imprinting after booster doses or reinfection was also demonstrated and identified as a major determinant of immunological responses to repeated antigen exposures. Considering that a small domain of the SARS‐CoV‐2 Spike protein, the receptor binding domain (RBD), is the major target of neutralizing antibodies and concentrates most viral mutations, the following text aims to provide insights into the ongoing debate over the best strategies for vaccine boosters. We address the relevance of developing new booster vaccines that target the evolving RBD, thus focusing on the relevant antigenic sites of the SARS‐CoV‐2 new variants. A combination of this strategy with immunofusing and computerized approaches could minimize immune imprinting, therefore optimizing neutralizing immune responses and booster vaccine efficacy.

## 1 SARS, MERS, and SARS‐CoV‐2

In the first two decades of the present century, three coronaviruses (CoV) emerged that caused severe respiratory infections in humans. SARS‐CoV emerged in late 2002 in Guangdong Province, China, and caused severe acute respiratory syndrome (SARS). Another coronavirus, MERS‐CoV, emerged in the Middle East in 2012 and caused Middle East Respiratory Syndrome (MERS). While these outbreaks generated concern, none of them developed into a pandemic. SARS‐CoV‐2 first appeared near the end of 2019 in Wuhan, China [1]. It is not known precisely how the first humans were infected, but all currently available evidence points to a zoonotic origin, since the virus is closely related to coronaviruses found in bats [2]. In 2020, SARS‐CoV‐2 infection led to a pandemic (COVID‐19) probably as a consequence of several factors including its higher transmissibility among humans, the ability for asymptomatic and presymptomatic transmission, global mobility, socioeconomic factors, environmental stability of viral particles, and variability in public health interventions [3, 4].

**FIGURE 1:**
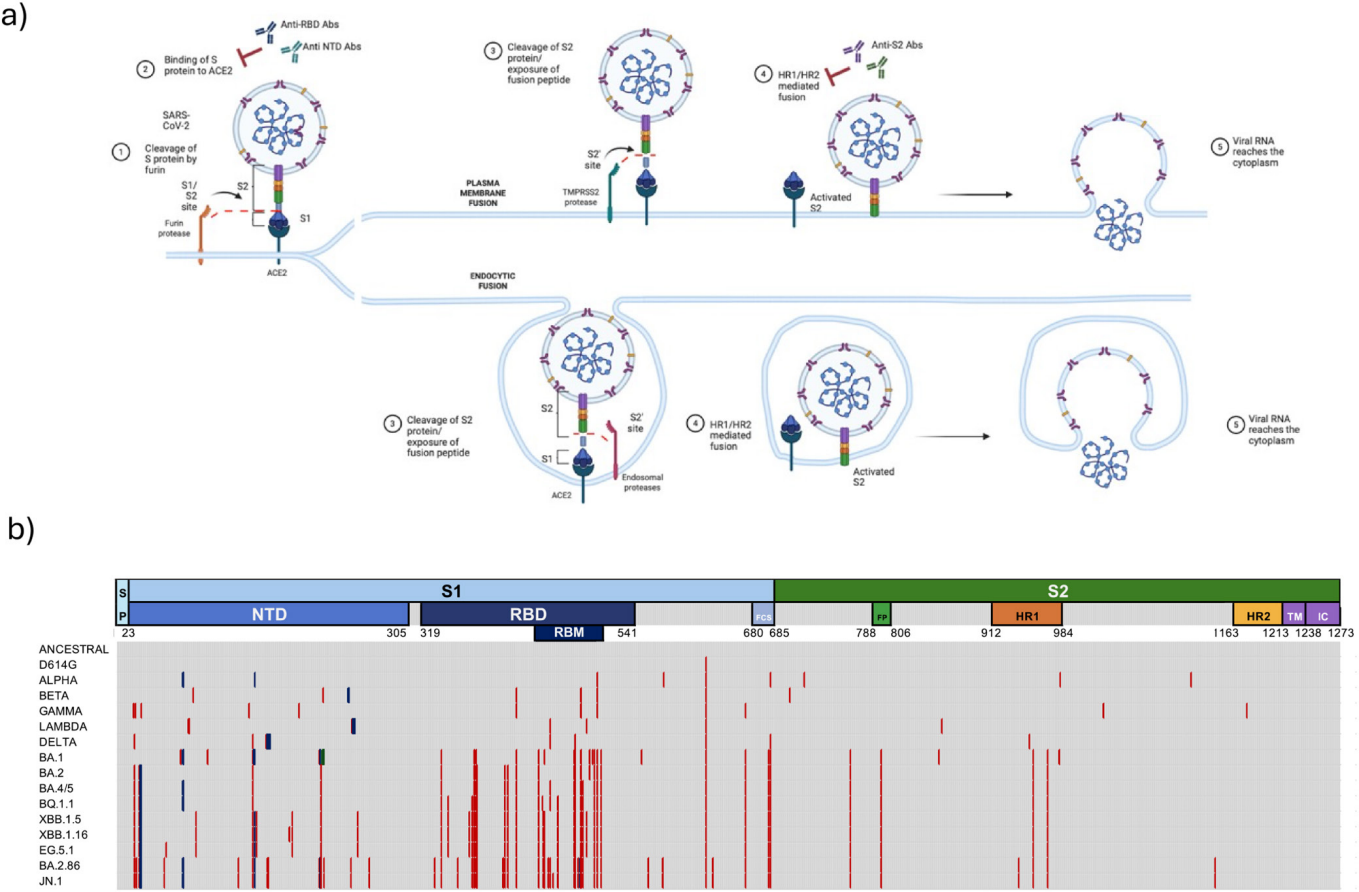
Function and evolution of the Spike protein of SARS‐CoV‐2. (a) Schematic representation of SARS‐CoV‐2 entry mechanisms. The SARS‐CoV‐2 viral particle exposes the Spike protein on its surface. The protein is processed by furin to yield S1 and S2 subunits. The S1 subunit binds to the ACE 2 receptor on the cell surface, a process blocked by neutralizing anti S1 (RBD or NTD) antibodies. Further processing of the Spike protein can occur at the plasma membrane by the protease TMPRSS2, which cleaves the S2 protein to expose the fusion peptide that will be inserted in the host membrane. The HR1 and HR2 regions of S2 will coordinate membrane fusion (a process sensitive to neutralizing anti‐S2 antibodies) allowing the access of viral RNA to the cytoplasm. Alternatively, in the absence of efficient processing at the cell surface, the virus will be endocytosed, and the S2 subunit will be processed by endosomal proteases allowing for endosomal membrane fusion and infection. (b) Evolution of the Spike sequence through the pandemic. The full‐length spike sequence is shown, numbers correspond to amino acid positions according to the ancestral sequence, and main regions are color‐coded as depicted in Panel (a). Most relevant pandemic variants are shown on the left. For each variant red symbols correspond to mutations, blue symbols correspond to deletions, and green symbols correspond to insertions. Of note, the highest mutation rate is observed in the RBD and the core receptor binding motif (RBM) regions. FCS: furin cleavage site; FP: fusion peptide; HR1: Heptad Repeat 1; HR2: Heptad Repeat 2; IC: intracellular domain; NTD: N‐terminal domain; RBD: receptor binding domain; RBM: receptor binding motif; SP: signal peptide; TM: transmembrane domain.

The sequence and structure of the virus were rapidly identified [5], and as for other CoVs, the virus was found to contain four main structural proteins: a central nucleocapsid (N), an external large trimeric spike (S), a smaller membrane (M), and envelope (E) proteins embedded in the envelope lipid bilayer. Based on previous vaccine developments for SARS‐COV‐1 and MERS, and because the S protein mediates the virus entry, this protein was considered the optimal target to be used as an antigen in vaccine development. The S protein is composed of two functional subunits, including the S1 and S2 subunits. The S1 subunit consists of the N‐terminal domain (NTD) and the receptor binding domain (RBD) and mediates the binding of the virus to target cells. The S2 subunit contains the fusion peptide (FP), the heptad repeat 1 (HR1), the central helix (CH), the connector domain (CD), the heptad repeat 2 (HR2), the transmembrane domain (TM), and the cytoplasmic tail (CT) and mediates virus/cell membrane fusion promoting subsequent infection steps (Figure 1) [6].

## 2 SARS‐CoV‐2 Infection and COVID‐19 Pathophysiology

In 2004, Hamming et al. [7] established the Angiotensin Converting Enzyme‐2 (ACE2) protein as the functional receptor for SARS‐CoV infection. Similarly, SARS‐CoV‐2 entry also occurs after the binding of the S protein to ACE2 through the RBD, a motile domain located on the apex of the S1 subunit (Figure 1a). After this initial binding step, the S protein undergoes proteolytic cleavage by host proteases, either at the plasma membrane or after endocytosis, leading to the exposure of the fusion peptide located on the S2 subunit of the trimer. This event triggers the activation of the membrane fusion mechanism and allows for the intracellular release of the viral RNA (Figure 1a).

Infection elicits an early coordinated immune response which can rapidly clear the virus [8]; however, in the absence of appropriate immune responses to halt viral replication, SARS‐CoV‐2 spreads in different tissues leading to the upregulation of the p38 MAPK (mitogen‐activated protein kinase) pathway and, ultimately, the development of a “cytokine storm” that can lead to multiple organ failure [9, 10]. The widespread expression of the ACE2 receptor in epithelial cells across various tissues and organs makes them susceptible to SARS‐CoV‐2 infection. This also elucidates the virus's potential for causing tissue damage and organ failure, contributing to understanding the mechanisms of SARS disease's pathological manifestations [11].

## 3 Vaccines, Immune Responses, and Duration of Protection

A wide range of vaccine approaches were developed at an unprecedented speed after the identification of SARS‐CoV‐2. Several vaccine platforms, including mRNA, genetically engineered viral vectors, recombinant proteins, and inactivated viruses, were successfully developed with the most successful given emergency use authorization within the first year of the pandemic [12].

All these tested vaccines were based on the sequence of the ancestral virus strain (from the first infected patients) and, when focused on the S protein, mostly used as immunogen an engineered version of the S protein stabilized in the prefusion conformation (before the S protein fuses with the host cell) [13]. These immunogens were delivered through viral vectors, mRNA, or recombinant protein formulations. These vaccines demonstrated high efficacy in preventing symptomatic SARS‐CoV‐2 infection, 94% (95% CI, 89 to 97%) for the vaccine mRNA‐1273 [14] and 95% (CI, 90 to 98%) for vaccine BNT1626b [15]. However, these impressive efficacy values were progressively reduced several months after vaccination, even before variants of concern arose. Despite this observation, vaccines maintained effectiveness against severe disease or death [16, 17, 18]. This dichotomy was linked to the more stable dynamics of cytotoxic responses compared to humoral responses [19, 20], which have been associated with protection against infection [21]. Earlier studies in patients who recovered from severe COVID‐19 or mild COVID‐19 convalescent individuals demonstrated a rapid decline and further stabilization of neutralizing antibodies [22] sustained by long‐lived B cells directed against SARS‐CoV‐2 S [23]. These observations suggested that the strong humoral immune response to the infection would not be sustained for long periods, a situation that was confirmed in the ensuing months and years. Similarly, the robust antibody response triggered by modified mRNA vaccines declined over time [24]. An analysis of eight SARS‐CoV‐2 vaccines, based on data from 33,418 patients, revealed that all eight vaccines produced through different platforms lost most of their protective effect against symptomatic infection between 8 and 11 months after the first dose [25]. Waning humoral immunity was still occurring following booster immunizations with mRNA vaccines, as the durability of serum antibody responses improved only transiently [26, 27]. Two systematic reviews and meta‐analyses further confirmed the findings on waning protection of the ancestral strain‐based vaccines developed against SARS‐CoV‐2. In a meta‐analysis, including 50 studies, Rahman et al. [28] evaluated the protection of third‐ and fourth‐dose mRNA vaccines against the SARS‐CoV‐2 Omicron subvariant and its sublineages. Booster doses provided substantial protection against severe COVID‐19 outcomes for at least 5–6 months, although the exact duration of protection remained unclear. Another meta‐analysis [29] reviewed the waning of post‐vaccination neutralizing antibody titers against the ancestral strain or Omicron BA.1. Waning occurred at similar rates across different vaccination regimens and prior‐infection status strata. Infected and uninfected vaccinated individuals showed similar waning although higher responses were associated with hybrid immunity. Waning was faster for Omicron BA.1, with most individuals not reaching protective neutralizing antibody levels. In addition, some studies have reported that the waning of vaccine effectiveness against hospitalization was more pronounced in high‐risk populations, such as older adults, immunosuppressed patients, and those with comorbidities and was associated with lower initial values [30].

While the initial laboratory assessment of vaccine efficacy was based on antibody responses, cellular immunity, mediated by CD8 T cells, has proven to be instrumental in providing durable and effective protection against SARS‐CoV‐2 severe disease [31]. Although COVID‐19 vaccines can induce S‐specific cytotoxic T‐cell responses [32, 33, 34], N and M targeted T‐cell responses seem to be critical for protection against infection, severe disease, and durability of T‐cell response [35, 36]. As COVID‐19 vaccination can induce both cellular and humoral responses, they ensure a multi‐layered defense against SARS‐CoV‐2, contributing significantly to reduced transmission, severity, and mortality.

## 4 Mutations. Escape From Humoral Neutralizing Antibody Responses

SARS‐CoV‐2 started to mutate very soon after it was identified. The Spike protein D614G mutation was first detected in January 2020 and became dominant by early spring 2020 [37]. After that, an increasing number of mutations were described [38]. Most of these mutations affected the conformation of the S protein and, more specifically, the RBD (Figure 1b). Five SARS‐CoV‐2 variants of concern (VOC) have been classified by the WHO: Alpha (B.1.1.7), Beta (B.1.351), Gamma (P.1), Delta (B.1.617.2), and Omicron (B.1.1.529) [39]. Although vaccination campaigns and previous infections reduced the risk associated with new variants, the nowadays predominant Omicron subvariants continue to evolve, accumulating mutations in diverse sublineages at a very high rate [40].

Mutations accumulated across the whole viral genome impacting several viral functions, such as the control of innate immunity and adaptation to a new host, but the highest variability was found in the S gene, particularly the RBD [41], and more specifically in the receptor binding motif (RBM) directly responsible for the ACE2/Spike contact (Figure 1b). The relationship between RBM/RBD mutations and escape of neutralizing antibodies was observed since the first variants [42]. This was fully confirmed with the emergence of the Omicron variants, which were resistant to antibodies elicited by previous infection with earlier variants or by the standard two‐dose vaccination schedule using ancestral strain vaccines [41, 43]. Not surprisingly, these mutations rendered the virus resistant to most therapeutic monoclonal antibodies previously developed [44]. Consistently, several studies have shown that neutralizing activity is mostly associated with antibodies targeting the RBD region, representing 80% of total neutralizing activity after natural infection and > 90% after whole Spike mRNA vaccination [45]. Although most RBD‐specific antibodies are only active against a limited number of variants, the RBD seems to be the major target of broad SARS‐CoV‐2 neutralizing antibodies elicited by vaccination [46], likely due to the presence of conserved epitopes that allow broad cross‐protection from particular anti‐RBD antibodies [47, 48, 49].

In summary, mutations in the RBD have a critical impact on viral infectivity, transmissibility, and escape to neutralizing antibodies. Alterations of the RBD in mutated forms of SARS‐CoV‐2 have been instrumental in understanding the virus's ability to spread and have also helped to provide the scientific rationale for developing potent RBD‐based vaccine strategies.

## 5 Confronting New SARS‐CoV‐2 Variants. Learning From Past Boosting Strategies

**FIGURE 2:**
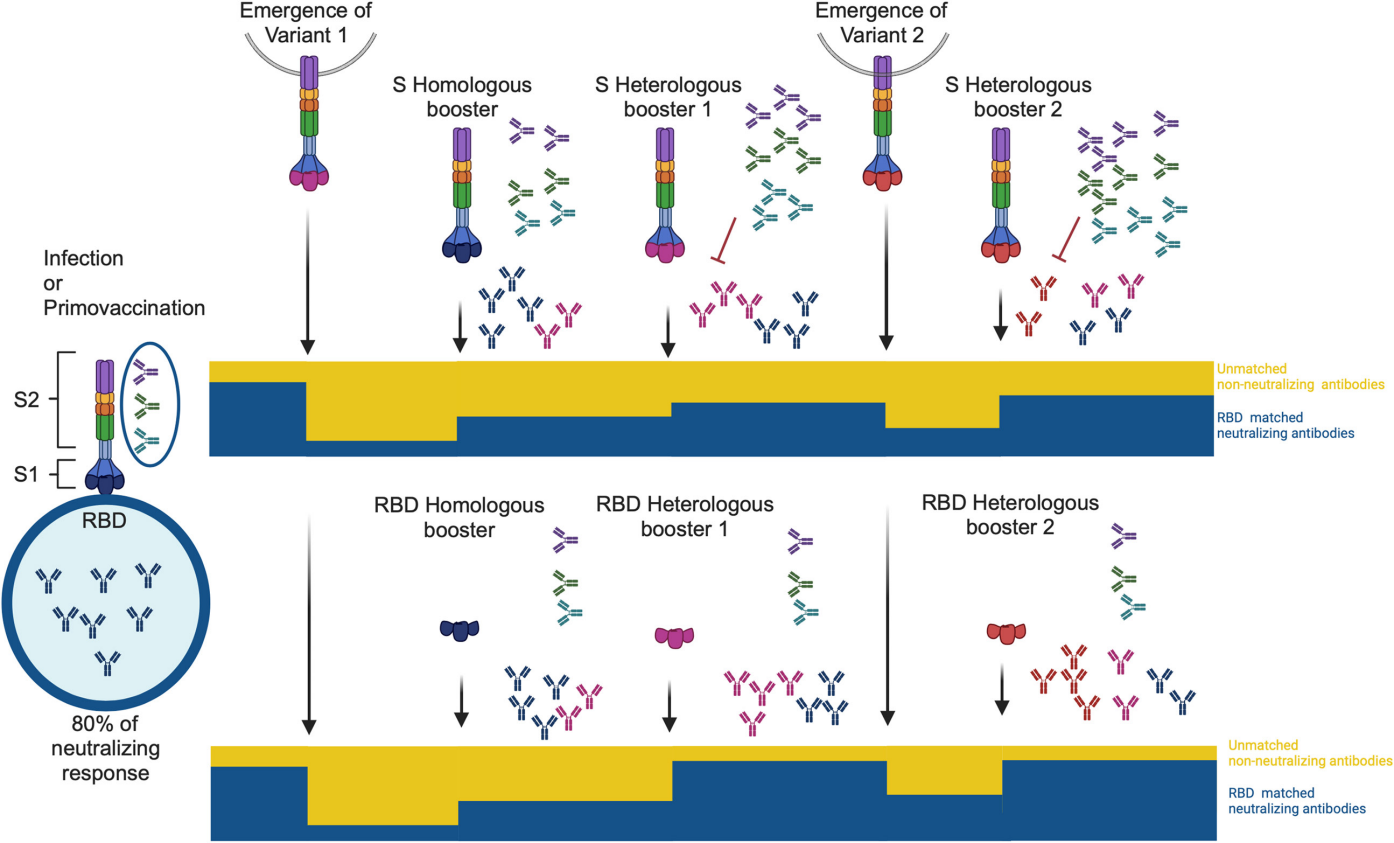
Hypothetical antibody evolution after repeated booster vaccination with full‐length Spike or RBD‐specific immunogens. Both primo vaccination and natural infection induce a wide repertoire of antibodies mainly focused on the RBD, in general, with a protective neutralizing activity. The emergence of new variants (variant 1 with a different RBD sequence) reduces the neutralizing capacity that can be restored by homologous vaccination (ancestral sequence) or more efficiently by heterologous vaccination with variant 1–based vaccine. Continued viral evolution will again reduce neutralization capacity that will need further boosters. The use of full‐length Spike vaccines would lead to the expansion of non‐RBD antibodies and would hinder the development of RBD‐specific de novo‐specific antibodies (upper panel). In contrast, immunization with RBD immunogens should avoid the expansion of non‐RBD specific B cell clones and promote a better response to new epitopes.

Initial booster strategies became necessary to face Delta and Omicron variants in 2021. These strategies used the available ancestral sequence‐based vaccines and were effective in diversifying and increasing antibody titers conferring protection against early Omicron variants [43]. However, further viral evolution led to the recommendation of new bivalent vaccines based on a combination of ancestral and subsequent Omicron variants (BA.1 or BA.5) [50]. However, the introduction of such bivalent boosters was impacted by previous immunological imprinting and likely by antigenic distance between the ancestral strain and Omicron variants, resulting in poorer specific responses to new epitopes, and suboptimal effectiveness of booster immunizations [51]. Repeated mRNA vaccinations with the ancestral S sequences could result in higher immunogenicity of conserved regions of the S protein and could prevent the production of antibodies against the relevant RBD epitopes exposed in new variants (Figure 2). Wang et al. [52] observed that a fourth dose of bivalent mRNA vaccine encoding ancestral and Omicron BA.5 spikes or a fourth dose of the monovalent ancestral vaccine alone similarly improved neutralizing antibody titers against Omicron mutations, suggesting a limited impact of new vaccine formulations. Multiple analyses, including antigenic mapping, indicated that the ancestral spike contained in bivalent vaccines limited vaccine effectiveness due to the immune imprinting caused by repeated vaccination with original vaccines [53]. Due to the imprinting effect and the unlikely reemergence of ancestral variants, the ancestral strain was no longer included when updating COVID‐19 vaccines in 2023, when a monovalent vaccine using the prevalent circulating variant (XBB.1.5) was approved [54]. Similar findings indicating persistent immune imprinting were communicated by Tortorici et al. [55] in studies on XBB.1.5 monovalent vaccines, though a recent study has demonstrated that the XBB.1.5‐adapted booster can partially overcome the persistent imprinting of SARS‐CoV‐2 immunity by wild‐type based vaccines [56]. Furthermore, a recent study reported that repeated Omicron exposures could be necessary to alleviate ancestral vaccination‐induced immune imprinting and generate broad neutralization responses both systemic and in nasal mucosa [57].

The latest recommendations for the 2024 COVID‐19 vaccination campaign have also been based on monovalent vaccines presenting the newest circulating variants, either JN.1 or KP.2 Spike antigens [58, 59, 60].

## 6 The Future of Boosters: Targeting the Receptor‐Binding Domain (RBD)

Considering that a high proportion (95%) of the population in Western countries is seropositive for the S protein through vaccination, infection, or both [61]. The current debate is focused on the best strategies for confronting new emerging SARS‐CoV‐2 variants through vaccination in such a highly antigen‐experienced population [58, 62]. Important questions remain about the use of monovalent or multivalent vaccines of adapted full Spike or domain‐specific antigens.

The mRNA and recombinant full‐length S protein vaccines proved successful in generating protective immunity; however, the strong dependence of neutralizing capacity on the highly variable RBD [63] and waning immunity remain as limitations. Continuous viral evolution will require further vaccinations to maintain protective immune responses. Therefore, the full‐length S vaccines will face immunological imprinting potential depending on the antigenic variations between former and emerging variants selected for vaccination [57]. In addition, for mRNA platforms [64], the possibility of an IgG class switch towards the IgG4 subclass after repeated boosters has been described with a subsequent impaired immune capacity of the antibodies generated [65, 66].

The RBD as vaccine antigen in any available vaccine platform can focus the neutralizing immune response in highly variable regions, minimizing imprinting and triggering higher variant‐specific responses. A wide array of RBD immunogens have shown safety and immunogenicity in humans and have been approved in different countries (Table 1). Although mRNA‐based RBD vaccines have been described, limited comparison to full‐length Spike vaccines is available [67, 68]. In contrast, more information is available on the immune response to adjuvanted recombinant RBD‐based vaccines, whose protection against severe infection is sustained up to 1 year [69]. Furthermore, in head‐to‐head comparisons to the mRNA vaccine (Supporting Information), adjuvanted RBD yielded superior levels of neutralizing antibodies both in the short‐term (14 days post‐vaccination) and the long‐term (6 months) [70, 71], suggesting better maintenance of neutralizing antibodies in primed individuals.

**TABLE 1:** Representative examples of approved vaccines aimed at the RBD motifs [17].

Vaccine	Developer/manufacturer	SARS‐CoV‐2 RBD composition	Countries approved	Expression platform	Adjuvant
Abdala	Center for Genetic Engineering and Biotechnology (CIGB)	RBD	6	*P. pastoris*	Aluminum hydroxide gel
Bimervax	HIPRA	Dimeric RBD (heterodimer)	27	CHO	SQBA
Corbevax	Biological E Ltd.	RBD	2	*P. pastoris*	Alhydrogel with CpG 1018
Indovac	PT Bio Farma	RBD	1	*P. pastoris*	Aluminum and CpG 1018
Noora vaccine	Baqiyatallah University of Medical Sciences	RBD		*E. coli* BL21 DE3	Alum adjuvant
Recombinant SARS‐CoV‐2 vaccine	National Vaccine and Serum Institute	Trimeric RBD (heterologous tri‐RBD)	1	CHO cells	Aluminum hydroxide
Soberana 02 Soberana Plus	Instituto Finlay	Conjugated tetanus toxoid‐RBD RBD dimer (homodimer)	42	CHO cells	Aluminum hydroxide gel
SKYCovione	SK Bioscience Co. Ltd.	Self‐assembling, two‐component nano‐particles displaying RBD	1	Expi293F cells	AS03
V‐01	Livzon Mabpharm Inc.	RBD fused with interferon alpha at N‐terminus and dimerized by human IgG1 Fc at C‐terminus	1	CHO cells	Aluminum hydroxide
Zifivax	Anhui Zhifei Longcom	Dimeric RBD (homodimer)	4	CHO cells	Aluminum hydroxide

Adjuvanted recombinant RBD vaccines offer significant manufacturing advantages compared to other vaccine platforms. These include the potential for manufacturing a large number of temperature‐stable doses with reduced expenses for materials, labor, and overheads and enhanced scalability, leading to more affordable vaccines and offering the opportunity for broader distribution, acceptance, and accessibility [72]. Moreover, the recombinant protein vaccine‐based technology has been evolving to become a faster and more efficient platform. It now allows to update the vaccines to the constantly emerging SARS‐CoV‐2 variants in less than 4 months, from the new sequence to commercial‐scale manufacturing. All these advantages, together with a potentially better acceptance of protein‐based vaccines in the population [73], make the RBD‐based recombinant protein vaccines a potential platform for worldwide accessibility to SARS‐CoV‐2 immunizations that could be administered within already available infrastructures.

The limitations of RBD‐subunit antigens include their lower immunogenicity compared to the full Spike protein in terms of cellular responses. However, this limitation may be less significant for booster doses, which aim primarily to elevate neutralizing antibody levels rather than preexisting T‐cell responses. As mentioned earlier in the text, preexisting T‐cell responses are stable and broad after vaccination [31, 36]. Therefore, the limited T‐cell immunogenicity of the RBD as compared to the full‐length S is likely to have a minimal impact on overall vaccine effectiveness and can be compensated by the elicitation of new variant‐specific T‐cell responses. Additionally, adjuvanted vaccines that utilize multimeric RBD arrangements have the potential to enhance both humoral and T‐cell responses [70, 74]. For instance, a vaccine featuring a recombinant RBD fusion dimer has demonstrated promising results in both preclinical studies and clinical trials [75, 76] with promising results, and various other multimeric presentations are currently under development (Table 1).

The highly immunogenic antigen formulations used in the RBD‐based platforms must be accurately defined to ensure the maximal efficacy of these vaccines. A comprehensive mapping with structural information of SARS‐CoV‐2 S protein and/or RBD in mutated variants is vital for the efficient design of future boosters. The overwhelming availability of plasma, sera, and antibodies obtained during the COVID‐19 pandemic has facilitated the high‐resolution mapping of the RBD structure in original and mutated variants of the SARS‐CoV‐2 [63, 77, 78]. Immunofocusing [79] could be applied to precise antigen engineering for the development of RBD‐based subunit vaccines of highly variable viruses such as SARS‐CoV‐2 [80], circumventing problems with imprinting observed with other platforms [79, 81]. An adequate interaction between high‐resolution mapping methods with immunofocusing technologies opens possibilities for high‐precision immunogen engineering recreating molecular conformations of the RBD [82]. Theoretically, such structures could be designed to rationally develop epitope‐focused vaccines via antigen reorientation [83].

Artificial intelligence (AI) is revolutionizing vaccine development by enabling diverse applications, including predicting immune responses, designing nanobody binders, optimizing vaccine doses, and improving vaccine stability [84, 85]. AI is being applied to computerized models that can rapidly predict multiple protein configurations, advancing candidates with theoretically more aggressive mutations [86]. AI and machine learning concepts can be introduced at various stages of vaccine development. AI tools are being implemented for modeling, designing, and improving the resolution of antigens, but also for predicting immune response from several protein sequences, predicting changes in protein structures, or discovering adjuvants [87]. AI tools will help design mRNA vaccines that are more potent and stable [88] . AI‐based models also show promise for identifying antigenic targets for B‐cell and T‐cell immunogens that could boost antibody production [89, 90] or exploring therapeutic targets for other infectious diseases [91]. In addition, AI‐based approaches will also play an important role in addressing logistics, manufacturing, storage, safety, and effectiveness issues associated with COVID‐19 vaccine candidates [92].

## 7 Conclusions

Current booster SARS‐CoV‐2 vaccination strategies have significant potential for improvement compared to those used during the early stages of the COVID‐19 pandemic. Since booster shots will likely be necessary to guard against current JN.1 and its variants in the short term and to future variants of concern of the virus [51]. The next generation of vaccines will need to preferentially stimulate immunity towards epitopes linked to immune evasion rather than just enhance immunity to more specific conserved S domains (such as the S2 region) in individuals who have been previously immunized (Figure 2).

Selection of variant(s) for new boosters should consider the neutralization profile of vaccine candidates against circulating variants. Therefore, just as with influenza, there should be the option not to update the vaccine composition if, based on a correlate of protection, it could be demonstrated that a vaccine continues to generate a neutralizing response against variants circulating in the following season. Unfortunately, there are no defined antibody titers for vaccine protection.

In this repeated vaccination scenario, RBD‐based vaccines are positioned to perform better than vaccines targeting the whole SARS‐Cov‐2 S protein. Engineered RBD antigens tailored for precise immunofocusing to aid in the prevention of immune imprinting and to facilitate the development of broad‐spectrum vaccines against diverse coronaviruses.

mRNA and recombinant protein platforms can be rapidly adapted to RBD‐engineered immunogens, but the latter's favorable cost‐efficient vaccine manufacturability (production and storage) can contribute to broader distribution, improving global health outcomes.

Determining optimal booster strategies will require thoroughly assessing and conducting comparative studies of available vaccines. In this regard, preclinical data and immunobridging could be helpful to make quick public health decisions for future booster campaigns.

## Author Contributions

**Julià Blanco:** conceptualization, writing – original draft. **Benjamin Trinité:** writing – review and editing. **Joan Puig‐Barberà:** conceptualization, writing – original draft.

## Conflicts of Interest

J.B. reports institutional grants from Grifols, HIPRA, NESAPOR Europe, and MSD and honoraria from HIPRA, NESAPOR Europe, and La Caixa Foundation. Unrelated to the submitted work, J.B. was the founder and shareholder of AlbaJuna Therapeutics S.L. J.P.B. has received honoraria from HIPRA, Novavax, Pfizer, and Seqirus/CSL for providing expert advice and participating in research and conferences.

### Peer Review

The peer review history for this article is available at https://www.webofscience.com/api/gateway/wos/peer‐review/10.1111/irv.70076.

## Supporting information


**Data S1.** Supporting information.

## Data Availability

No datasets have been generated in this work.
