# Supplementary material for: Rethinking Optimal Immunogens to Face SARS‐CoV‐2 Evolution Through Vaccination
Source: Influenza Other Respir Viruses. 2025 Jan 28;19(1):e70076. doi: 10.1111/irv.70076 (PMC11773156; doi:10.1111/irv.70076)
Supplement: Supplementary file 1 — Data S1. Supporting information. [file IRV-19-e70076-s001.pdf]

# Rethinking optimal immunogens to face SARS-CoV-2 evolution through vaccination.

## Supplementary material

### Head-to-head comparisons of recombinat RBD and full-Spike mRNA vaccines

The RBD as vaccine antigen in any available platform can concentrate the neutralizing immune response in highly variable regions, minimizing imprinting and triggering higher variant-specific responses. A wide array of RBD immunogens have shown safety and immunogenicity in humans and have been approved in different countries (**Table 1**). Although mRNA-based RBD vaccines have been described, limited comparison to full-length Spike vaccines is available [1] [2]. In contrast, more information is available on the immune response to adjuvanted recombinant RBD-based vaccines, which have been shown to be sustained up to 1 year [3]. Furthermore, in head-to-head comparison to mRNA vaccine, adjuvanted RBD yielded superior levels of neutralizing antibodies at the short-term (14 days after vaccination) and the long-term (6 months) [4,5], suggesting less waning effect over time.

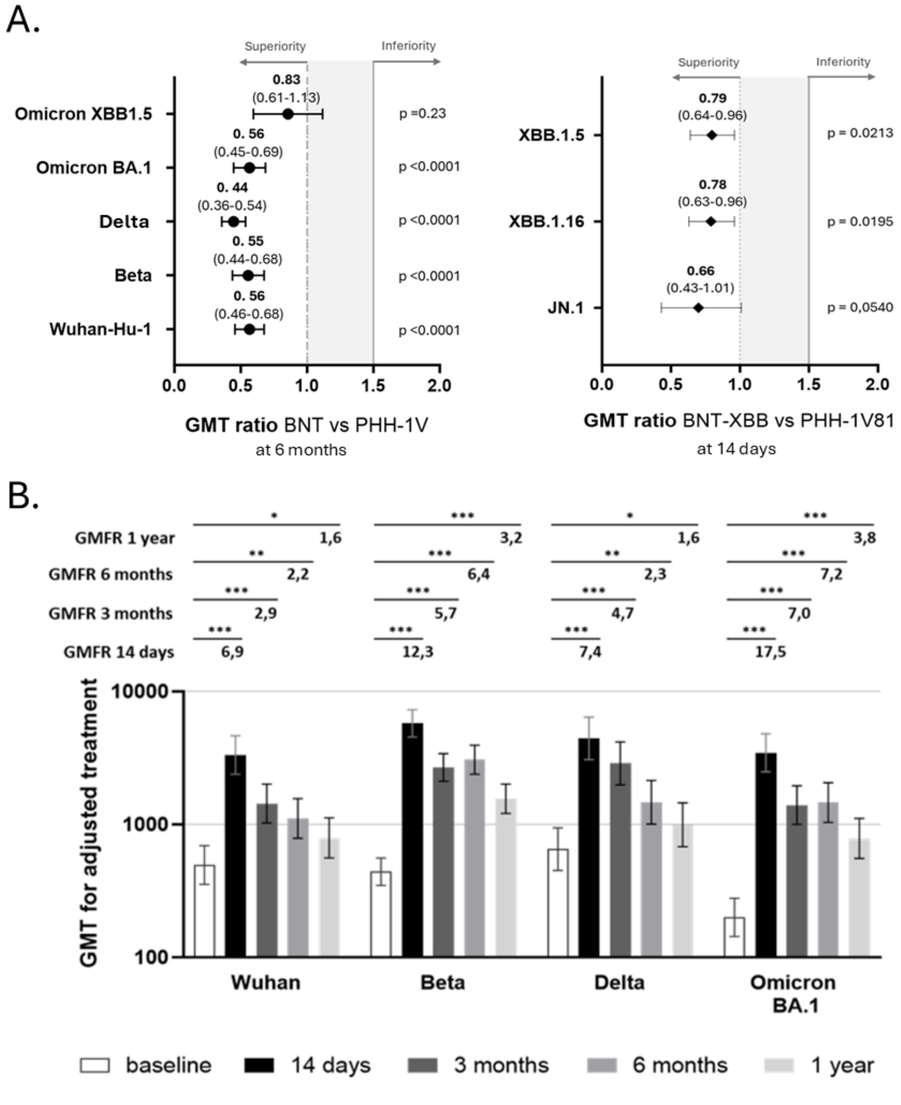


**Immunogenicity response of RBD-based adjuvated recombinant vaccine.**

**(A.) Non-inferiority analysis of humoral immunogenicity between mRNA-based vaccine and RBD-based vaccine against SARS-CoV-2 variants.** Forest plot for GMT ratio (95% CI) between mRNA-based vaccine and RBD-based vaccine. The solid line indicates

the non-inferiority limit of the trial (NIm = 1.5) and the dashed line indicates the superiority limit. p-values from GMT ratio = 1.0). (**left panel**) Analysis between BNT162b2 vs PHH-1V at 6 months (Adapted from Corominas J. *et al* [5]); (**right panel**) Analysis between BNT162b2-XBB 1.5 vs PHH-1V81 at 14 days (Adapted from Lopez-Fernandez M.J. *et al* [4]

**(B.) Neutralizing antibody levels against SARS-CoV-2 variants after booster with PHH-1V over time.** Representation of Mean GMT for adjusted treatment with the 95% CI (graphics) and mean GMFR (Upper numbers) from baseline against SARS-CoV-2 Wuhan, Beta, Delta and Omicron BA.1 variants in overall subjects (n= 235) at baseline (White) and Days 14 (Black), 91 (Dark grey) , 182 (Grey) and 365 (Light grey) post-boost. *** p< 0.0001; ** p< 0.001; * p<0,01 difference from GMFR ratios= 1. (Expanded data from Natalini S. *et al* [3])

## References


1 Liu, X. *et al.* Safety and superior immunogenicity of heterologous boosting with an RBD-based SARS-CoV-2 mRNA vaccine in Chinese adults. *Cell Res* **32**, 777-780, doi:10.1038/s41422-022-00681-3 (2022).

2 Nolan, T. M. *et al.* Interim results from a phase I randomized, placebo-controlled trial of novel SARS-CoV-2 beta variant receptor-binding domain recombinant protein and mRNA vaccines as a 4th dose booster. *EBioMedicine* **98**, 104878, doi:10.1016/j.ebiom.2023.104878 (2023).

3 Natalini Martínez, S. *et al.* Safety and immunogenicity of a phh-1v booster dose after different prime vaccination schemes against COVID-19: Phase III clinical trial final results up to one year. *medRxiv*, 2024.2005.2014.24307343, doi:10.1101/2024.05.14.24307343 (2024).

4 López Fernandez, M. J. *et al.* Immunogenicity and safety of an Omicron XBB.1.16 adapted vaccine for COVID-19: Interim results from a randomized, controlled, non-inferiority clinical trial. *medRxiv*, 2024.2004.2019.24306064, doi:10.1101/2024.04.19.24306064 (2024).

5 Corominas, J. *et al.* Humoral and cellular immune responses after 6 months of a heterologous SARS-CoV-2 booster with the protein-based PHH-1V vaccine in a phase IIb trial. *medRxiv*, 2024.2002.2001.24302052, doi:10.1101/2024.02.01.24302052 (2024).
